# A New Hæmostatic

**Published:** 1897-06

**Authors:** 


### A New Haemostatic.

Dr. Froham, a dentist of Berlin, has recently given to the profession the results of his experiments on Ferripyrin, and states that it has great haemostatic properties. Its application to more than 100 stubborn cases of bleeding gave excellent results. Post-haemorrhaga seldom resulted, and when it did further application caused permanent arrest. There are no painful after-effects and it yields perfect coagulation. Ferripyrin is a combination of one part ferric chloride, three parts antipyrin and five parts water. It is a dark red liquor and can be purchased at the drug stores. It has an agreeable taste and is readily applied. In case of a lower extraction employ a small spoon and direct a sparing amount of the mixture into the socket; in the event of an upper case apply a saturated pellet of cotton to the socket. It gives immediate relief, and one application usually suffices.—*Journal fur Zahnheilkunde.*

